# Yield, Physicochemical Properties, and Fatty Acid Profile of Vegetable Oil Extracted From Seed Coats of *Strychnos pungens* Soler. Fruits Collected in Chikomba, Zimbabwe

**DOI:** 10.1155/2024/3257054

**Published:** 2024-10-26

**Authors:** Clemence Zimudzi, Godfrey E. Zharare

**Affiliations:** ^1^Department of Biological Sciences and Ecology, University of Zimbabwe, PO Box MP167, Mount Pleasant, Harare, Zimbabwe; ^2^Department of Agriculture, University of Zululand, Private Bag X1001, KwaDlangezwa, KwaZulu Natal, South Africa

**Keywords:** oleic acid, screw press, seed coat oil, *Strychnos pungens*

## Abstract

The study examined *Strychnos pungens* Soler. (family LOGANIACEAE) fruit as a potential source of vegetable oil. Ripe fruits collected from a forested site in Zimbabwe were processed to determine the partitioning of fresh and dry fruit biomass. The oil was extracted from the seed coat using a hand–operated screw press, and its physiological properties were analyzed. Seeds contributed the most to the fresh weight of the fruit, followed by the shell and pulp. The seed coat was a significant component of the seeds. The seed coat, but not the pulp of the fruit, was found to contain screw press-extractable oil, the yield of which was substantial, amounting to around 39% of the dry weight of the seed coat. The oil was found to have a high free fatty acid content and a moderate iodine value (83 gI_2_/100 g), indicating a degree of unsaturation. Furthermore, the oil contained carotenoids and tocols, which serve as antioxidants that help to protect the oil from oxidation. The oil had a high content of monounsaturated oleic acid (78.3%), which is known for its stability and health benefits. The low levels of saturated and polyunsaturated fatty acids make it a high oleic oil. The volatile profile of the oil included compounds with pleasant fruity aromas that enhance its flavour and fragrance. The results highlighted the need for waste management strategies if *S. pungens* is industrialized as an oil crop. Significant waste, including shells, pulp, cake residue, and seed kernels, would need proper handling and valorisation. In summary, the research showed that *S. pungens* has the potential to be a valuable source of high-quality vegetable oil with good oxidative stability and health benefits, primarily due to its high content of oleic acid and antioxidant compounds.

## 1. Introduction

*Strychnos pungens* Soler. (family LOGANIACEAE) is one of the six *Strychnos* species of large fruits endemic to Africa. The other species are *S. spinosa*, *S. cocculoides*, *Strychnos madagascariensis* (*S. unoccua*), *S. gerrardii*, and *S. lucens* [1–3]). Among these, *S. pungens* is more closely related to *S. madagascariensis* (*S. unoccua*), *S. gerrardii,* and *S. lucens*, which together are members of the Densiflorae (Duvign.) section of the Loganiaceae family [4–6]. It is distinguishable among *Strychnos* species by its tough, elliptical, smooth leaves with a spiny apex [3, 7, 8]. All these *Strychnos* species have edible fruits. However, they have remained largely neglected and underused. This is more so for *S. pungens* and *S. lucens,* which do not seem to be processed or incorporated into traditional foods unlike the other *Strychnos* species. For *S. pungens*, this is despite that it has a wide distribution pattern in central and southern Africa [9]. Although it is widely abundant in sub-Saharan African countries; including South Africa, Democratic Republic of Congo, Zimbabwe, Zambia, Tanzania, southern Angola, Malawi, Tanzania, Botswana, and Namibia [3, 10], the fruit is only consumed fresh. No attempts have been reported to cultivate the fruit tree. However, it is often left standing in rural areas when the forest is cleared for cultivation [11].

The fruits of *S. pungens* are smooth and round with a hard woody shell that is approximately 5–15 cm in diameter [3, 12]. The unripe fruits are bluish green and turn yellow or orange after ripening [12]. The pulp changes from white in unripe fruits to yellow/orange in ripe fruits [12]. Unlike *S. cocculoides* and *S. spinosa* whose fruit pulp becomes gelatinous upon ripening, the pulp of *S. pungens* remains firm upon fruit ripening, similar to that of *S. madagascariensis*. Each fruit contains many (20–100) yellowish-white seeds that have a hard coat and are usually flat, glossy, irregular, curved, and always embedded in fleshy yellowish pulp [3, 8, 13]. The pulp of the ripe fruit is nonpoisonous and edible. Mwamba [3] has dismissed the presence of strychnine in both the pulp and seeds of *S. pungens*. The provenances in Zimbabwe, Namibia, and Botswana have fruit that is considered by the locals to be of good quality and is highly appreciated [10]. On the contrary, the fruit of the provenance in South Africa is not as popular because it has an unpleasant smell and a generally bitter taste [10].

The plant does not tolerate water logging. Because of this, it is found growing on deep sands in Zimbabwe, Namibia, Botswana, Zambia, the Republic of Congo, and Cameron and on ledges, rocky slopes, or at the base of stony kopjes in Witwatersrand and Magaliesburg in South Africa [10].

Although abundant in southern and central Africa, *S*. *pungens* has not been seriously considered for commercialization or industrialization due to a lack of knowledge about the useful products that can be developed from it. Recently, Zharare et al. [14] demonstrated that *S. madagascariensis*, a closely related species to *S. pungens*, has an appreciable amount of vegetable oil in its seed coat, amounting to approximately 42%, highlighting its potential as an alternative source of vegetable oils. Studies on *S. madagascariensis* oil have shown that it is rich in unsaturated fatty acids, particularly oleic acid (18:1) and linoleic acid (18:2). These fatty acids are known for their beneficial health effects, including cardiovascular protection and anti-inflammatory properties. The oil also contains smaller amounts of saturated fatty acids such as palmitic acid (16:0) and stearic acid (18:0), which contribute to its stability and shelf life. *S. madagascariensis* forms part of the growing list of oleaginous African indigenous plants, including marula (*Sclerocarya birrea*), baobab (*Adansonia digitata*), and Ximenia species, each with a particular fatty acid composition. The commercialization of these plants significantly contributes to rural economies where the plants naturally grow, highlighting the importance of exploring underutilized plant species for their oil yield potential and fatty acid composition. Our study on *S. pungens* oil adds to this expanding body of knowledge on vegetable oils from indigenous plants.

## 2. Materials and Methods

The ripe fruits of *S. pungens* were collected from four trees at a forested site in the Chikomba area (http://maps.google.com/?q=-18.59399,30.90742) (latitude: −18,59399; longitude: 30,90742; altitude: 1381 m) along the Harare-Masvingo road in Zimbabwe. This was done after obtaining permission from the landowners. Bright orange fresh-looking ripened fruits that were still attached to the plants were harvested, placed in polypropylene woven bags, and taken to the laboratory for processing. The fruits were processed upon arrival at the laboratory.

### 2.1. Biomass per Fruit and Its Partitioning Between Fruit Pulp, Seeds, and Shell

To determine the biomass partition between the fruit components, 15 fruits were randomly selected per tree for each of the four harvested trees. Each fruit was individually weighed, the shell cracked open, and the seeds were manually extracted by hand. These were counted and their fresh weight was determined. The pulp was removed from the shell with a spoon and the weights of both the shell and pulp were determined per fruit. These fruit parts were dried in an oven at 60°C until a constant weight was reached, and their dry weights determined.

### 2.2. Partitioning of Fresh Seed Biomass Into Seed Components

**Figure 1:**
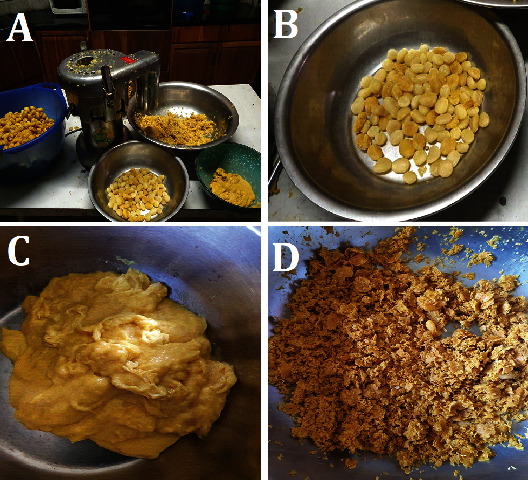
Pictograms showing (A) the centrifuge juicer that was used to scrape the seed coat from (B) the kernels to obtain (C) the oil-containing seed coat puree and (D) the husks without oil.

For each of the four trees whose fruits were harvested, a quantity of ca. 2500–2800 g of seeds was obtained from fruits, and the seed coats were separated from the hard seed kernels using a centrifuge juice extractor (Gatto heavy–duty juice extractor model WFA3000) as described for *S. madagascariensis* by Zharare et al. [14]. The juicer separated the components of the seeds into (i) thick seed coat puree, fibrous seed coat tissue, and seed kernels (Figure 1). These were weighed and then dried in an oven at 60°C. There was not enough material from each tree to replicate.

### 2.3. Oil Extraction and Yield Determination

The dried sheets of seed coat puree were cut into small pieces to facilitate easy feeding into the inlet of a hand-operated screw press, as described by Zharare et al. [14]. Oil extraction was performed using this hand-operated screw oil press, which has a processing capacity of 3 kg of material per hour. For each extraction, 200 g of seed coat samples was used. The expressed oil was collected in a beaker and weighed immediately after extraction. The oil yield was calculated by expressing the weight of the extracted oil as a percentage of the initial 200 g of seed coat material. This percentage represents the oil yield for each extraction.

### 2.4. GC-MS Analyses of Volatile Compounds in Seed Coat Oil

#### 2.4.1. Sample Preparation

Approximately 0.25 mg of oil samples were placed into a CTC headspace vial. Five milliliters of a 12% alcohol solution (v/v) (pH = 3.5) were added, followed by 5 mL of a 20% sodium chloride (NaCl) solution. The mixture was vortexed to ensure thorough mixing. The headspace of the sample was then analyzed using solid phase microextraction (SPME)-GC-MS with a DVB/CAR/PDMS (gray) SPME fiber.

### 2.5. Determination and Quantification of Volatiles

The volatiles in the seed coat oil were determined by comparing their mass spectra with those in the NIST/EPA/NIH Mass Spectral Library. Retention indices were calculated based on a series of alkane standards run under the same conditions. The quantification of volatiles was carried out by integrating the peak areas of the identified compounds in the chromatograms. The relative amounts of each volatile compound were expressed as a percentage of the total peak area to provide a quantitative assessment of the volatiles present in the seed coat oil.

### 2.6. Chromatographic Separation

Analysis was performed on an automated Thermo Scientific TRACE 1310 gas chromatograph injection coupled to a TSQ 8000 MS/MS Triple Quadrupole. Chromatographic separation was achieved using a polar ZBWAXPlus capillary column (30 m, 0.25 mm ID, and 0.25 *μ*m film thickness). One microliter of the sample was injected in a 20:1 split ratio. The mass spectrometer operated in scan mode (35–650 m/z). The ionization source temperature was set at 250°C, and an emission current of 50 *μ*A was used with argon as collision gas.

### 2.7. Determination of Physicochemical Properties of Seed Coat Oil

The physiochemical parameters of the oil (peroxide value, free fatty acids (FFAs)/acid value, anisidine value, unsaponifiable matter, saponification value, iodine value, fatty acid profile, relative density, and refractive index) were determined according to the methods listed in Table 1. Details of the methods are provided as supporting information (Supporting Information S1). A brief description of the methods used for the determination of fatty acid profiles, carotenoids, and tocopherols is hereunder provided.

### 2.8. Carotenoid Content

The carotenoid content was determined using the Malaysian Palm Oil Board method MPOB-P2.6.2004 [16]. A 0.15 g sample was dissolved in iso-octane, and absorbance was measured at 446 nm to calculate carotenoid content using a formular outlined in Supporting Information S1.

### 2.9. Fatty Acid Profile

Fatty acids were derivatized to methyl esters, separated by gas chromatography with flame ionization detection, and identified using an external fatty acid methyl ester mixture (Supporting Information S1) [15].

### 2.10. Tocopherols and Tocotrienols

The tocols were measured by normal phase HPLC following the ISO 9936:2016 method [17]. The oil samples were dissolved in n-hexane, filtered, and analyzed using HPLC with fluorescence detection. Alpha-tocopherol was used as a reference for calculating the tocopherol and tocotrienol contents using the formular provided in Supporting Information S1.

## 3. Results

### 3.1. Fresh Biomass of the Fruit Components

The average fresh weight per fruit for the four trees sampled was similar, although within the tree the weight was somewhat variable ranging from 270 to 330 g (Table 2). The number of seeds per fruit was strikingly similar between the trees sampled. The seed number averaged at 28 per fruit. Between the fruit components assessed, the seed contributed the most to the fresh weight, ranging from 50.51% to 54%. (Table 2). The proportion of the fresh weight of the fruit contributed by the shell was between ca. 28.59% and 31.92%, while the fresh biomass in the pulp was almost half that of the shell. Unlike the cases of the fresh weight of the shell and the seed whose proportions did not differ significantly between the four trees sampled, that of the pulp varied significantly between the four trees. However, the variation was within a narrow range from 15.68% to 19.88%.

Using the trees as replicates, the seed coat was on average around 41% of the seed biomass, and the rest of the seed biomass was made up of seed kernels (Table 3). The seed coat consisted of a fluid oil-bearing layer underlain by a fibrous layer. The oil-bearing layer was ca. 71% of the fresh seed coat biomass with very small differences between the trees that were sampled. The rest of the fresh seed coat biomass was the fibrous base layer that enveloped each kernel.

### 3.2. Dry Biomass of Fruit Components

In terms of dry weight, the seeds contributed on average the most dry biomass (49%) of the fruit. Second, was the fruit shell, which on average contributed approximately 43% to the dry biomass of the fruit (Table 4). The pulp of the fruit contributed the least dry fruit biomass, accounting for only 7.72% of the fruit biomass. Between the seed components, the seed coat was ca. 32% of the dry seed mass, whilst the seed kernels (pips) formed 68% of the dry seed mass. The fibrous layer of the seed coat accounted for approximately 12.38% of the seed coat's dry biomass, while the oil-bearing layer made up about 19.29% of the total seed coat biomass (Table 5). Within the oil-bearing layer, oil comprised 39% of its biomass. This translated to 7.575% of the seed dry biomass per fruit and 3.74% of the whole dry fruit.

### 3.3. Physiochemical Composition of *S. pungens* Oil

An analysis of the physiological properties of *S. pungens* vegetable oil (Table 6) indicated that the oil was characterized by a fairly high FFA content that averaged 32 ± 5 g oleic acid/100 g of oil and an acid value that stood at 65 ± 10 mg KOH/g of sample on the one hand. On the other hand, the peroxide value of the oil was below the detection limit and its anisidine was relatively low at 4.43 ± 0.7. The saponification value was moderate (189.3 ± 3.4 mg KOH/g sample) and was accompanied by a fairly low unsaponifiable matter (2.49 g/100 g). The iodine value was also moderate (83.0 gI_2_/100 g). The oil had a carotenoid content of 3.8 ppm and a refractive index of 1.4662.

### 3.4. Fatty Acid Composition of *S. pungens* Oil

Eight fatty acids were identified in the oil (Table 7). Among these were two monosaturated fatty acids, of which cis oleic acid dominated the oil with a content of 78.30%. Eicosenoic acid was the other monosaturated fatty acid but was present only in trace quantities (0.85%). In the oil were four saturated fatty acids that included small quantiles of palmitic acid (9.70%) and stearic acid (2.21%) and tiny amounts of arachidic acid (0.58%) and lignoceric acid (0.37%). Linoleic (4.56%) and linolenic (2.80%) acids comprised the only polysaturated oil present in the oil.

### 3.5. Tocol Profile

The oil had a total of 171 mg/kg of tocols, which was equivalent to a vitamin activity of 63.3 mg/kg. This consisted of 58.0 mg/kg *α*-tocopherol, 10.7 mg/kg *β*-tocopherol, and 81 mg/kg *β*-tocotrienol (Table 8).

### 3.6. Volatile Compounds

Based on the NIST 95 and WILEY275 libraries for compound matches, 155 volatile compounds were detected in *S. pungens* oil. Among them, 21 volatile compounds had > 1% area (Table 9). A full list of volatiles obtained from *S. pungens* is provided as supporting information (Supporting Information S2).

## 4. Discussion

### 4.1. *S. pungens* Fruit as a Source of Vegetable Oil

*S. pungens* joins *S. madagascariensis* as a *Strychnos* species that contain extractable vegetable oil by screw press in the seed coat [14]. However, the oil content (39% by weight/dry by weight) of the seed coat of *S. pungens* in the present study was lower than that (40%–42%) obtained for *S. madagascariensis* by Zharare et al. [14]. Contrary to expectation, the pulp of the fruit did not produce extractable oil by screw press, a characteristic that was also observed for *S. madagascariensis*. It is difficult to explain why in both species the seed coat contains screw press-extractable oil, while the pulp surrounding the seed fails to yield screw press-extractable oil. This may be related to the different rewards that *Strychnos* species offer their seed dispersers. The bulk of the tissue internal to the fruit is made up of the seeds. The primary seed dispersers of the plant are mainly mammals which mainly eat the seeds whose kernels pass through the digestive tract undigested. Therefore, the oil in the seed coat could be seen as a reward.

Unlike the seed coat of *S. madagascariensis*, that for *S. pungens* does not naturally detach from the seed kernel upon drying but remains tightly bound to the seeds. This makes it difficult to separate the seed coat from the kernel in dried *S. pungens* seeds. In this regard, the most attractive pre-extraction option is to scrape the seed coat off the kernels of fresh seeds immediately after removal from the fruit and extract the oil using a screw press from the dehydrated puree of the seed coat. This was the option chosen in the present study. The seed coat of *S. pungens* was found to constitute ca. 41% and 15.63% of the fresh and dry biomass of the fruit, respectively. The seed coat had two distinct layers consisting of a fibrous nonoil-bearing layer on top of which was an oil-bearing layer containing ca. 39% dry wt/wt screw press extractable oil. Overall, the oil content of the dry fruit (3.74%) was much lower than that (8.13%) obtained by Zharare et al. [14] for *S. madagascariensis*. There are two possible reasons for this difference. First, the oil-containing seed coat which constituted 21% and 15.63% of the fresh and dry fruit of *S. pungens*, respectively, were less than the values of 27% and 19.36% obtained for the fresh and dry biomass of the fruit of *S. madagascariensis* by Zharare et al. [14]. Second, the seed coat of *S. pungens* had a substantial amount of fibrous layer that did not contain oil, and third, the oil content in the oil–containing seed coat layer (38%) was lower than that (42%) obtained for *S. madagascariensis* by Zharare et al. [14].

With the advent of industrializing *S. pungens* as an oil crop, a considerable amount of waste would be generated in the form of shells, pulp, cake residue, and seed kernels. In this respect, the extraction of oil from *S. pungens* fruit will account for 3.74% percent of the fruit dry biomass, leaving behind around 96.26% of the fruit dry mass as waste made up of approximately kernels (33.72%), pulp (7.72%), shells (42.95%), and seed coat residues (11.89%). This waste should be appropriately managed. Preferably, the path of action for managing the waste should be through an integrated biorefinery approach, whereby the seeds and the shells are used in making activated carbon/charcoal, for example. Consequently, there is a need to research ways to valorise these waste components into economic streams.

### 4.2. Fatty Acid Composition of *S. pungens* Seed Coat Oil

With regard to the composition of vegetable oils, the primary compounds of interest are the fatty acids present in triglycerides, the major of which are oleic acid, linoleic acid, and palmitic acid, among others. These fatty acids play an essential role in the determination of the nutritional properties, stability, and sensory attributes of vegetable oils. Again, as is the case with *S. madagascariensis* seed coat oil [14], the *S. pungens* seed coat oil has a very high monosaturated oleic acid content (78.30%) compared to the very low contents of saturated fatty acids (9.70%), namely, stearic acid (2.21%), arachidic acid (0.58%), and lignoceric acid (0.37%). The contribution of polysaturated fatty acids to the oil in terms of both the number and the quantity was even lower, consisting of 4.56% linoleic acid and 2.80% linolenic acid. This makes it a high-oleic oil. In this regard, *S*. *pungens* is in the same category as extra virgin olive oil whose oleic acid content accounts for up to 70%–80% of the oil [18]. Having a high content of oleic acids offers several important advantages to the oil, from enhanced stability and extended shelf life to potential health advantages and culinary versatility [18–21]. The underlying property of oleic acid that imparts these advantages is that it is a monounsaturated fatty acid, which makes it less susceptible than oils with higher levels of polyunsaturated fatty acids to oxidation and rancidity, resulting in a longer shelf life and a reduced risk of spoilage [21]. As a potential edible oil, the high oleic acid content is believed to make the oil withstand high temperatures without breaking or forming harmful compounds, allowing longer fry lifetimes and less frequent oil replacement [21–23]. Furthermore, high-oleic oils have a lower tendency to form polymers during heating, which means they are less likely to create sticky residues on cooking surfaces and food items. In terms of health, oleic acid is considered a healthy monounsaturated fat for the heart [18, 24]. Therefore, oils high in oleic acid have been associated with potential health benefits, such as reducing bad cholesterol levels (LDL cholesterol) and supporting cardiovascular health [18, 25]. It reduces the risk of cardiovascular diseases [25] and suppresses the tumorigenesis of inflammatory diseases [26]. In the review of the benefits of oleic acid, Lu et al. [18] have highlighted the following health benefits of oleic acid: inhibition of oxidative stress and inflammation, regulation of LDL and HDL levels, protection of vascular endothelial function, and alleviation of hypertension.

Due to its high thermal-oxidative stability and viscosity relative to other common fatty acids, monounsaturated fatty acids, such as oleic acid, also have significant industrial potential [27, 28]. Consequently, there has been an increasing interest in the use of plant oils with high oleic acid as a renewable raw material in the production of biolubricants and biodiesel [29]. In this regard, the high oleic acid content of *Strychnos* oils makes them good candidates as sources of oleic acid for cleavage processing of oleic acid to produce monomers of azelaic acid for nylon production [30].

### 4.3. FFA Content

As previously noted for *S. madagascariensis* oil [14], the oil extracted from the seed coats of *S. pungens* was found to have a high acid value (65 ± 10 mg KOH/g) and a high FFA (34.2 ± 5 g oleic acid/100 g oil). The reason for the high content of FFAs in the seed coat oils of the two *Strychnos* species is not yet clear. Zharare et al. [14] have hypothesized that the high content of FFAs emanates from the breakdown of fats by lipase during the ripening of the fruits [31, 32], whose activity is expected to increase together with the activities of other hydrolytic enzymes during the ripening of this climacteric fruit [32, 33]. The presence in the oil of a relatively high amount of glycerol (Table 9), a by-product derived from the breakdown of fats by lipase/lyases, supports this hypothesis. In addition, the low peroxide value (lower than the limit of quantification) of the oils excludes the oxidative deterioration of the oil as a source of FFAs. This is further supported by the moderately low anisidine value (4.43) obtained for the oil, a parameter generally associated with secondary oxidation. Whatever the reasons for the high FFAs in the oil, it is a significant issue that can cause several problems. In addition to being a health hazard for humans [34, 35], high levels of FFA in oil can lead to increased susceptibility of oils to oxidation, which causes the oil to become rancid faster [35, 36], leading to a shorter shelf life and rendering it unsuitable for consumption or commercial use. Rancidity not only affects the taste and aroma of the oil but also reduces its nutritional value [35]. An oil with high FFAs can develop an unpleasant taste and odour due to the formation of volatile compounds during oxidation and hydrolysis [34, 35]. Mitigating these problems may require a complicated refining process leading to increased costs and potential losses during production.

### 4.4. Iodine Value

The oil had a moderate iodine value of 83 gI_2_/100 g which was slightly above that (78.8–79 gI_2_/100 g) for *S*. *madagascariensi*s ([14]. The iodine value reflects the degree of unsaturation. The seed coat oils of both *S. madagascariensis* [14, 37] and *S. pungens* (in this study) contain > 78% monounsaturated fatty acids and, therefore, a somewhat moderate iodine value. The range of the iodine values for *S. pungens* and *S. madagascariensis* (78–83 gI_2_/100 g) is typical of nondrying oils (about 80 gI_2_/100 g), such as olive oil, used for soap making and in food products. Drying oils, such as linseed oil, used in the paint and varnish industry have relatively high iodine values of ca. 155–205 gI_2_/100 g [38], while semidrying oils, such as soybean oil, have intermediate iodine values of about 125–140 gI_2_/100 g [39, 40]. A high iodine value makes oils prone to rancidity when exposed to air, light, and heat, which reduces their storability, which requires special attention to storage and handling to prevent oxidation and rancidity [41]. Moderate values of iodine (70–100 gI_2_/100 g), such as those observed for *S. pungens* in this study and for *S. madagascariensis* in a previous study [14], represent moderate levels of unsaturation and impart a good balance between stability and flexibility in oil [42]. This is probably the reason why both oils have undetectable peroxide values and low anisidine values. The low peroxide and anisidine values also suggest that *Strychnos* oils are highly protected from oxidation, at least when freshly extracted.

#### 4.4.1. Saponification Value

The saponification value corresponds to the mass (in milligrams) of potassium hydroxide needed to neutralize free fatty acids and saponify esters contained in 1 g of material. It is an important parameter in various industries, particularly in soap and detergent manufacturing, as well as in the production of cosmetics and pharmaceuticals. In soap manufacturing, a high saponification value indicates that a smaller quantity of fat or oil is required to produce a given amount of soap. This can be advantageous as it leads to cost savings and more efficient production. In detergent manufacturing, the knowledge of the saponification value is crucial to formulating effective cleaning products. Higher saponification values can lead to stronger cleaning agents in certain cases. They can also be useful for formulating cosmetics and pharmaceuticals, as they help determine the properties and characteristics of the final product. Different oils and fats have varying saponification values, which can affect the texture and performance of creams, lotions, and other products. The saponification value of 189.3 mg KOH/g obtained in this study is comparable to that of most of the major oils used in the production of soaps such as avocado (187.5 mg KOH/g), cocoa butter (193.8 mg KOH/g), olive (189.7 mg KOH/g), palm (199.0 mg KOH/g), and sunflower (188.7 mg KOH/g) [43]. This shows that *S. pungens* oil also has potential in the manufacture of soaps and detergents.

#### 4.4.2. Antioxidant Potential of *S. pungens* Oil and Protection From Oxidation

The oil had two sources of protection against oxidation, namely, carotenoids and tocols [44–46], and. However, the carotenoid content was low, only 3.8 ppm (parts per million). This level of carotenoids in the oil can provide some protection against oxidation, but, in general, a higher concentration of carotenoids is more effective in providing antioxidant protection. Therefore, while having carotenoids at 3.8 ppm is beneficial and can contribute to the overall antioxidant capacity of the oil, this low level of carotenoids might not be enough to provide robust protection against oxidation on its own. Having a total antioxidant content that includes carotenoids and other antioxidants offers better protection against oxidation than oils with a single type of antioxidant [44, 47, 48]. Another class of potent antioxidants present in *S*. *pungens* oil was tocol. Commonly known as vitamin E [45, 49], they are a group of fat-soluble antioxidants that play a crucial role in protecting oils and fats from oxidation [45, 49, 50] and, thus, preventing rancidity and deterioration of the quality of the oil. They help neutralize free radicals and reactive oxygen species, preventing oxidative damage to the oil and extending its shelf life. In the present study, the oil was found to contain a total of 171 mg/kg of tocols comprising 58.0 mg/kg *α*-tocopherol, 10.7 mg/kg *β*-tocopherol, and 81 mg/kg *β*-tocotrienol (Table 9), which was equivalent to vitamin activity of 63.3 mg/kg. Combining carotenoids and a higher tocol content such as 171 mg/kg would be a more effective strategy to improve the oxidative stability of oil [48]. This means that the oil is less likely to undergo rancidity and develop off-flavours, making it suitable for a longer storage period and higher temperature cooking applications. Besides aiding in protecting oils from oxidation, tocols are a valuable source of vitamin E (in the form of tocols), which when consumed contributes to overall health and well-being due to its essentiality for human health and its known antioxidant and anti-inflammatory properties [51, 52]. Furthermore, vitamin E is believed to offer other various health benefits, such as supporting the immune system, promoting skin health, and protecting cells from oxidative stress [52]. Including oils with a good amount of tocols in the diet can help meet the body's vitamin E needs. Overall, the tocol content of 171 mg/kg in *S. pungens* signifies its fairly good antioxidant status.

### 4.5. Volatile Compounds Detected in *S. pungens* Oil

#### 4.5.1. Flavour Enhancement Compounds

Among the 155 compounds that were detected in the volatile profile of *S. pungens* oil, 21 of the compounds existed in appreciable amounts that were greater than 1.0% (Table 9). Six of them, namely, ethyl decanoate (7.80%), ethyl 2,4-trans,cis-decadienoate (6.00%), ethyl octanoate (5.07%), octanoic acid/(3.83%), ethyl 2-methylbutyrate (3.50%), hexanoic acid, and ethyl ester (2.53%), have a pleasant fruity aroma and are generally used as flavouring agents and fragrance enhancers. Ethyl decanoate was the most dominant volatile compound. This compound is commonly used in aromatherapy as an artificial aroma in various applications, including alcoholic drinks, fruit juices, and perfumes, due to its fruity and sweet odour [53, 54]. Ethyl 2,4-*trans*,*cis*-decadienoate is found in some oils, where it imparts a characteristic fruity and sweet aroma and a pleasant tropical fruit-like scent, often described as peachy or apricot like [55]. Ethyl octanoate is commonly found in various natural sources, including oils, which also contributes to its characteristic fruity scent [56]. Ethyl 2-methylbutyrate, hexanoic acid, ethyl ester (ethyl caproate), and propyl 2-methylbutanoate are esters with a fruity and sweet aroma that should contribute to the overall pleasant flavour and aroma profile of *S, pungens* oil.

*S*. *pungens* oil joins coconut oil in having an appreciable amount of octanoic acid (3.83% of total fatty acids) also known as caprylic acid. Analysis in a previous study [14] also showed that *S. madagascariensis* contains appreciable amounts of caprylic acid (3.4% of total fatty acids). The range of octanoic acid content in *Strychnos* oil in this study and in that of Zharare et al. [14] is however less than 5%–10% of total fatty acids observed for coconut oil [57]. The presence of octanoic acid in both *Strychnos* oils is expected to impart some pharmacological benefits to the oils. Octanoic acid is a saturated medium-chain fatty acid with eight carbon atoms. When present, its importance in vegetable oil lies in various aspects related to pharmacology [58]. In addition to contributing to the overall flavour and aroma profile of vegetable oils, octanoic acid possesses antibacterial [59], antifungal, and anti-inflammatory properties [60, 61]. These properties make octanoic acid a helpful remedy for many skin conditions. Thus, the application of *S. pungens* oil to the skin oil can be expected to help in managing skin yeast and bacterial infections and skin inflammation conditions due to the health benefits imparted above by octanoic acid. Furthermore, the antimicrobial properties of octanoic acid itself are an omen to the oils containing octanoic acid in that it helps to inhibit the growth of microorganisms in the oils, thus contributing to preserving the oil's quality and extending its shelf life by reducing the potential for microbial spoilage. In addition to its antimicrobial properties, octanoic acid is considered a healthy fatty acid and is part of the group of medium-chain triglycerides that have been associated with various potential health benefits, including improved metabolism, weight management, and cognitive function [62]. Given all these benefits of octanoic acid, it might be worthwhile to assess diversity in the octanoic acid content of *S*. *pungens* and other *Strychnos* oils and to identify provenances which may have high contents of octanoic acid in their oils.

#### 4.5.2. Undesirable Compounds

Eight of the volatile compounds with contents above 1% of the total volatile compounds detected are generally not found in vegetative oils. They included (i) hexanoic acid (caproic acid), (ii) 1,3-butanediol, (iii) 2,3-butanediol, (iv) heptane, (v) ethyl *trans*-2,*trans*-4-decadienoate, (vi) 2-butenal (crotonaldehyde), (vii) furfural, and (viii) 2-methylbutyric acid. Among these, the presence of hexanoic acid, 2-butenal, and furfural in the oil is a source of concern. Hexanoic acid (also called caproic acid) is a saturated six-carbon fatty acid with a strong, pungent, and rancid odour. It is occasionally found in some oils in small amounts, for example, coconut oil and palm oil. Its presence in oils can have several implications. When present in oils, it can contribute to off-flavours and odours, negatively impacting the oil's sensory qualities. It is one of the volatile compounds formed during the oxidative degradation of polyunsaturated fatty acids in oils through the lipoxygenase pathway [63, 64]. Therefore, the presence of hexanoic acid can be an indication of the early stages of oil rancidity, which is the process of oil deterioration due to oxidation. 2-Butenal is found in fruits and vegetables, for example, tomato juice and strawberry aroma [65]. It is an eye, skin, and mucous membrane irritant [66]. Nevertheless, it has many industrial uses, including pharmaceutical, food, and perfumery [67].

In the context of vegetable oils, furfural is generally not expected to be present at significant levels [68]. Rather, its presence in oils is generally undesirable, as it can contribute to off-flavours and off-odours, negatively impacting the sensory quality of the oil [68]. Its presence in vegetable oil could be the result of heat during oil extraction [69].

#### 4.5.3. The Significance of Glycerol in the Volatile Compound Profile

The oil of *S. pungens* had glycerol (glycerine) in its volatile compound profile, accounting for 4.96% of the total volatile compounds. This was less than 6.1% observed for *S. madagascariensis* [14]. In general, the presence of glycerol in vegetable oils is not a concern, as it is a natural and harmless component. Rather, its presence in the oil is beneficial. It is a crucial component of vegetable oils that plays an important role in their structure and stability in applications that involve oil emulsions [70, 71]. This is because glycerol has emulsifying properties [70], which means that it can help stabilize oil–water mixtures. In food applications, this is useful for creating stable emulsions, such as salad dressings, mayonnaise, and various sauces. Because of its hygroscopic property, glycerol can attract and retain moisture. Thus, if *S. pungens* oil is used in cosmetic skin applications, the presence of glycerol in the oil should help to maintain the balance of moisture of the skin. In general, the presence of glycerol in *S. pungens* oils is expected to enhance the functionality, stability, and versatility of the oil in a wide range of industrial, food, and cosmetic applications, particularly those involving water–oil emulsions [70].

## 5. Conclusions

*S. pungens* was found, as is the case with *S*. *madagascariensis*, to contain screw press-extractable oil in its seed coat. However, the fruit pulp does not produce screw press-extractable oil. This difference might be related to the evolutionary adaptation of seed dispersal mechanisms and rewards offered to seed dispersers.

The seed coat of *S. pungens* tightly binds to the seed kernel, making separation difficult, which requires immediate processing after fruit removal to extract the oil efficiently. Additionally, the seed coat consists of two layers, with an oil-bearing layer containing approximately 39% screw press-extractable oil.

Industrialization of *S. pungens* as an oil crop would generate considerable waste, including shells, pulp, cake residue, and seed kernels. Proper waste management, possibly through an integrated biorefinery approach, is essential to utilize these waste components effectively.

The oil of the S. *pungens* seed coat is high in oleic acid, like extra virgin olive oil. This high oleic acid content provides various advantages such as enhanced stability, longer shelf life, and potential health benefits, making it suitable for culinary and industrial applications.

The oil has a high acid value and a high content of free fatty acids, possibly due to breakdown by lipase during fruit ripening. High levels of free fatty acids can lead to oxidation and rancidity, which negatively affects the quality and shelf life of the oil.

*S. pungens* oil exhibits moderate iodine and saponification values, indicating a significant level of unsaturation and making it a viable candidate for use in soap and detergent manufacturing. Oils and fats with moderate saponification values are often used in the production of soaps and detergents, as they balance hardness and lathering properties.

The oil of *S. pungens* contains various volatile compounds, including undesirable and flavour enhancer compounds. The presence of certain compounds such as octanoic acid may impart pharmacological benefits, while others such as hexanoic acid and furfural could lead to off-flavours and odours. The presence of glycerol in the oil improves its functionality, stability, and versatility in various industrial, food, and cosmetic applications, particularly in emulsions. In general, the *S. pungens* fruit shows potential as a source of vegetable oil, although careful consideration of its composition and properties is necessary for effective industrialization and utilization.

## Figures and Tables

**Table 1:** Methods used for physiochemical analyses of *S. pungens* oil, where AOCS is the American Oil Chemists' Society, MPOB is the Malaysian Palm Oil Board Test Methods, and ISO is the International Organization for Standardization.

**Parameter**	**Analytical method used**	**Reference**
Peroxide value	AOCS Cd 8-53	AOAC [15]
Free fatty acids/acid value	AOCS Cd 3d-53	AOAC [15]
Anisidine value	AOCS Cd 18-90	AOAC [15]
Unsaponifiable matter	AOCS Ca-6a-40	AOAC [15]
Saponification value	AOCS Cd 3-25	AOAC [15]
Iodine value	AOCS Cd 1c-85	AOAC [15]
Fatty Acid profile	AOCS Ce 2-66	AOAC [15]
Carotenoid content	MPOB P2.6:2004	MPOB [16]
Tocopherols	ISO 9936:2016	ISO [17]
Relative density	Guy–Lussac pycnometer	Not applicable
Refractive index	Atotago PAL-RI refractometer	Not applicable

**Table 2:** Variations in whole fruit fresh weight and in the proportions of fresh weights invested in the seeds, shells, and pulp of *S. pungen*s fruits (data are means of 15 fruits).

**Fruit part**	**Tree 1**	**Tree 2**	**Tree 3**	**Tree 4**	**Means**	**LSD**	**CV%**
Fruit fresh weight (g)	270	330	343	317	315	NS	11
Pulp (%)	15.68	19.88	18.70	15.95	17.55	2.850	12.2
Shell weight (%)	30.32	28.59	30.79	31.92	30.41	NS	17.1
Seed weight (%)	54.00	51.53	50.51	52.13	52.04	NS	10.6
Seed number per fruit.	24	29	29	29	28	NS	12.5

**Table 3:** The proportion of fresh seed biomass and seed coat biomass.

**Seed parameter**	**Kernels as % of fresh seed biomass**	**Seed coat as % of fresh seed biomass**	**Oil-bearing puree as % of fresh seed coat biomass**	**Fibrous layer as % of fresh seed coat biomass**
Tree 1	57.96	42.04	71.47	30.05
Tree 2	58.65	41.35	69.25	30.63
Tree 3	59.54	40.46	70.32	29.76
Tree 4	60.32	39.68	71.4	27.93
Mean	59.12	40.89	71.36	29.09
LSD_0.05_	NS	0.82	1.04	0.46

**Table 4:** Percentage of dry matter partitioned between the fruit and seed components of *S. pungens.*

**Tree**	**Fruit DM partitioned between fruit components (%)**			**Seed DM partitioned between seed components (%)**		**Seed coat dry matter peritonised between seed coat components**	
	**Seeds**	**Pulp**	**Shells**	**Kernels**	**Seed coat**	**Fibrous layer**	**Oil bearing layer**
1	50.49	7.09	42.42	67.34	32.65	12.90	19.76
2	49.32	9.36	41.32	69.11	30.88	13.51	17.38
3	50.85	5.19	43.97	69.77	30.23	10.21	20.02
4	46.70	9.23	44.07	67.10	32.90	12.89	20.01
Mean	49.34	7.72	42.95	68.34	31.67	12.38	19.29

**Table 5:** Percentage of oil extracted from the oil-bearing layer of the *S. pungens* seed coat using a screw press.

**Sample**	**Oil yield (% wt/dry wt)**
Extraction 1	39.83
Extraction 2	39.96
Extraction 3	38.02
Mean	39.27

**Table 6:** Physiochemical analysis of oil extracted from dried *Strychnos pungens* seed coats.

**Sample**	**Result**	**Uncertainty**	**LOQ**
Peroxide value (meq O_2_/kg)	Lower than LOQ		0.81
Acid value (mg KOH/g)	65	± 10	0.112
Free fatty acids (g oleic acid/100 g)	32.4	± 5.0	0.056
Anisidine value	4.43	± 0.73	0.52
Unsaponifiable matter (g/100 g)	2.49	0.44	0.24
Saponification value (mg KOH/g)	189.3	± 3.4	N/A
Iodine value (gI_2_/100 g)	83.0	N/A	N/A
Relative density^a^ (20°C)	0.910	N/A	N/A
Refractive index^a^ (20°C)	1.4662	N/A	N/A
Carotenoid content (ppm)	3.8	N/A	N/A

^a^Not Sanas accredited.

**Table 7:** Fatty acid composition of *Strychnos pungens* oil extracted from seed coats of fruits harvested from Chikomba in Zimbabwe.

**Fatty acid**	**%(Wt)**	**Uncertainty**
C14:0 myristic acid	Lower than LOQ	Not applicable
C16:0 palmitic acid	9.70	± 1.10
C16:1 palmitoleic acid	Lower than LOQ	
C18:0 stearic acid	2.21	± 0.11
C18:1 cis oleic acid	78.30	± 2.3
C18:2 cis linoleic acid	4.56	± 0.13
C18:3 n3 linolenic acid	2.80	± 0.20
C20:0 arachidic acid	0.58	± 0.12
C20:1 eicosenoic acid	0.85	± 0.16
C22:0 behenic acid	Lower than LOQ	
C24:0 lignoceric acid	0.37	± 0.13

**Table 8:** Tocols present in *S. pungens* seed coat oil.

**Tocopherol/tocotrienol** ^a^ ^b^	**Tocol content mg/kg**	**Uncertainty**
*α*-Tocopherol	58.00	± 5.4
*α*-Tocotrienol	Lower than LOQ	
*β*-Tocopherol	10.70	± 1.1
*γ*-Tocotrienol	Lower than LOQ	
*β*-Tocotrienol	81.00	± 12.0
*γ*-Tocotrienol	Lower than LOQ	
*δ*-Tocopherol	Lower than LOQ	
Total	149.70	± 17.0
Vitamin E activity (*α*-TE)^a^^c^)	63.30	

^a^Not Sanas accredited.

^b^ *α*-Tocopherol was used as the only standard for the calculations.

^c^Calculations of vitamin E activity: individual homologues are quantified, and the level is converted to RRR *α*-T equivalents (*α*-TE). One *α*-TE is equal to 1 mg *α*-tocopherol, 0.50 mg *β*-tocopherol, 0.10 mg *γ*-tocopherol, 0.03 mg *δ*-tocopherol and 0.30 mg *α*-tocotrienol.

**Table 9:** Volatile compounds from *S. pungens* seed coat oil with an area greater than 1.0%.

**Retention time (mins)**	**Compound**	**% area**
16..21	Ethyl decanoate	7.18
18.51	Ethyl 2,4-trans,cis-decadienoate	6.00
13..56	Ethyl octanoate	5.07
23..82	Glycerol	4.96
20..69	Octanoic acid	3.83
13.63	Acetic acid	3.63
6.49	Ethyl 2-methylbutyrate	3.50
18.49	Hexanoic acid	2.83
10.35	Hexanoic acid, ethyl ester	2.53
15.43	1,3-Butanediol	2.53
12.93	Nonanal	1.40
14.96	2,3-Butanediol	1.56
6.04	2-Butenal	1.37
13.93	Furfural	1.30
1.79	Heptane	1.31
8.43	Propyl 2-methylbutanoate	1.26
1.62	Hexane	1.22
18.36	Ethyl *trans*-2,*trans*-4-decadienoate	1.15
23.05	Decanoic acid	1.12
31.65	Hexadecanoic acid = palmitic acid	1.10
16.53	2-Methyl-butyric acid	1.03

## Data Availability

Details of materials and methods used for physicochemical characterization and fatty acid profile analyses of oil extracted by screw press from *Strychnos pungens* are provided in Appendix A. Also, a full list of volatiles obtained from *S. pungens* is provided as supporting information in Appendix B.

## Supporting Information

Supporting Information 1 S1. Details of materials and methods used for physicochemical characterization and fatty acid profile analyses of oil extracted by screw press from *Strychnos pungens*.

Supporting Information 2 S2. A list of volatile compounds detected in oil extracted by screw press from *Strychnos pungens.*
