# Supplementary material for: Yield, Physicochemical Properties, and Fatty Acid Profile of Vegetable Oil Extracted From Seed Coats of Strychnos pungens Soler. Fruits Collected in Chikomba, Zimbabwe
Source: J Lipids. 2024 Oct 26;2024:3257054. doi: 10.1155/2024/3257054 (PMC11531360; doi:10.1155/2024/3257054)
Supplement: Supporting Information 2 — S2. A list of volatile compounds detected in oil extracted by screw press from Strychnos pungens. [file 3257054.f2.docx]

**Supplementary S2. A list of volatile compounds detected in oil extracted by screw press from *Strychnos pungens.***

| **Retention time (mins)** | **Compound** | **Area%** |
| --- | --- | --- |
| 1.62 | Hexane | 1.22 |
| 1.74 | 3-Methylhexane | 0.048 |
| 1.79 | Heptane | 1.31 |
| 2.14 | Propanal | 0.015 |
| 2.19 | Octane | 0.056 |
| 2.28 | Acetone | 0.076 |
| 3.70 | Ethanol | 0.219 |
| 4.24 | Ethyl isobutyrate | 0.057 |
| 4.47 | 3-Methylbutanal | 0.186 |
| 5.51 | Alpha-pinene | 0.166 |
| 6.04 | 2-Butenal | 1.37 |
| 6.49 | Ethyl 2-methylbutyrate | 3.50 |
| 6.85 | Ethyl 3-methylbutyrate | 0.054 |
| 7.09 | Hexanal | 0.069 |
| 7.35 | Isobutyl isobutyrate | 0.020 |
| 7.73 | Isobutyl alcohol | 0.057 |
| 8.06 | Propyl butyrate | 0.051 |
| 8.19 | trans-2-Pentenal | 0.046 |
| 8.24 | Butyl methacrylate | 0.035 |
| 8.43 | propyl 2-methylbutanoate | 1.26 |
| 8.60 | butyl 2-methylpropanoate | 0.019 |
| 8.77 | Propyl isovalerate | 0.021 |
| 8.87 | beta-Myrcene | 0.011 |
| 8.95 | trans-2-Butenoic acid ethyl ester | 0.023 |
| 9.12 | Alpha-terpinene | 0.006 |
| 9.33 | 2-Heptanone | 0.041 |
| 9.36 | n-Heptanal | 0.038 |
| 9.43 | Hexanoic acid, methyl ester | 0.025 |
| 9.45 | pyridine | 0.025 |
| 9.53 | Limonene | 0.108 |
| 9.64 | Dodecane | 0.021 |
| 9.71 | beta-Phellandrene | 0.113 |
| 9.90 | (R)-(+)-3-Methylcyclopentanone | 0.030 |
| 10.04 | Butyl butyrate | 0.034 |
| 10.25 | 2-Pentylfuran | 0.016 |
| 10.35 | Hexanoic acid, ethyl ester | 2.53 |
| 10.39 | Ethyl tiglate | 0.582 |
| 10.54 | Heptadecane | 0.013 |
| 10.72 | Amyl alcohol | 0.079 |
| 10.92 | Para-cymene | 0.140 |
| 11.11 | m-Ethyltoluene | 0.022 |
| 11.14 | 2-Methylbutyl 2-methylbutyrate | 0.033 |
| 11.27 | n-Octanal | 0.205 |
| 11.46 | Tridecane | 0.045 |
| 11.49 | Hydroxyacetone | 0.062 |
| 11.79 | Propyl hexoate | 0.656 |
| 11.86 | Isopropyl tiglate | 0.922 |
| 12.02 | Ethyl n-heptanoate | 0.081 |
| 12.07 | 6-methyl-5-hepten-2-one | 0.011 |
| 12.16 | n-hexyl isobutyrate | 0.027 |
| 12.20 | ethyl E-2-hexenoate | 0.099 |
| 12.24 | 2-Ethylfuran | 0.040 |
| 12.32 | Isobutyl hexanoate | 0.092 |
| 12.36 | n-Ethylidenebutylamine | 0.032 |
| 12.42 | Isobutyl tiglate | 0.057 |
| 12.51 | 1,4-Dimethyl-2-ethylbenzene | 0.006 |
| 12.84 | 3-Ethylpyridine | 0.054 |
| 12.87 | Octanoic acid, methyl ester | 0.070 |
| 12.93 | Nonanal | 1.40 |
| 13.00 | Tetradecane | 0.142 |
| 13.05 | (E,E)-2,4-Hexadienal | 0.125 |
| 13.21 | Butyl hexanoate | 0.087 |
| 13.26 | Hexyl butyrate | 0.049 |
| 13.42 | hexyl 2-methylbutanoate | 0.103 |
| 13.56 | Ethyl octanoate | 5.07 |
| 13.63 | Acetic acid | 3.63 |
| 13.83 | Heptanol | 0.035 |
| 13.93 | Furfural | 1.30 |
| 14.10 | Ethyl cis-4-octenoate | 0.103 |
| 14.34 | trans,trans-2,4-Heptadienal | 0.209 |
| 14.38 | Formic acid | 0.760 |
| 14.50 | 2-Acetylfuran | 0.012 |
| 14.52 | Ethyl sorbate | 0.012 |
| 14.68 | propyl octanoate | 0.748 |
| 14.75 | Benzaldehyde | 0.358 |
| 14.89 | Ethyl pelargonate | 0.225 |
| 14.96 | 2,3-Butanediol | 1.56 |
| 15.13 | ethyl E-2-octenoate | 0.922 |
| 15.43 | 1,3-Butanediol | 2.53 |
| 15.54 | 4-Cyclopentene-1,3-dione | 0.203 |
| 15.61 | 1,2-Propanediol | 0.171 |
| 15.64 | Decanoic acid, methyl ester | 0.362 |
| 15.81 | 4-Aminostyrene | 0.178 |
| 16.01 | Valeric acid | 0.317 |
| 16.13 | gamma-Butyrolactone | 0.787 |
| 16.21 | Ethyl decanoate | 7.18 |
| 16.27 | trans-2-Decenal | 0.303 |
| 16.42 | Furfuryl alcohol | 0.857 |
| 16.50 | Ethyl cis-4-decenoate | 0.865 |
| 16.53 | 2-Methyl-butyric acid | 1.03 |
| 16.69 | ethyl 3-hydroxyhexanoate | 0.337 |
| 16.77 | p-Menth-1(7)-en-2-one | 0.045 |
| 16.81 | Ethyl cis-4-decenoate | 0.067 |
| 16.86 | 2-Cyclohexen-1-one | 0.050 |
| 17.09 | 8-Heptadecene | 0.085 |
| 17.18 | propyl decanoate | 0.338 |
| 17.28 | 3,4-dimethyl-2,5-Furandione | 0.198 |
| 17.33 | 3-Butenoic acid | 0.792 |
| 17.53 | Iso butyl decanoate | 0.261 |
| 17.62 | Ethyl trans-2-decenoate | 0.624 |
| 17.68 | 2-Butenoic acid | 0.223 |
| 17.85 | Methyl salicylate | 0.723 |
| 17.91 | Ethyl phenylacetate | 0.261 |
| 18.02 | Methyl trans-2-cis-4-decadienoate | 0.113 |
| 18.07 | delta-Hexalactone | 0.394 |
| 18.23 | Ethyl salicylate | 0.008 |
| 18.36 | Ethyl trans-2,trans-4-decadienoate | 1.15 |
| 18.42 | 3-Acetylpyridine | 0.065 |
| 18.43 | Cinnamaldehyde | 0.057 |
| 18.49 | Hexanoic acid | 2.83 |
| 18.51 | Ethyl 2,4-trans,cis-decadienoate | 6.00 |
| 18.71 | Guaiacol | 0.046 |
| 18.75 | exo-2-Hydroxycineole | 0.018 |
| 18.81 | Phenylacetic acid propyl ester | 0.071 |
| 19.19 | Methyl 5-methylsalicylate | 0.068 |
| 19.28 | Phenylethyl alcohol | 0.193 |
| 19.38 | dihydro-5-pentyl-2(3H)-Furanone | 0.025 |
| 19.50 | 2-phenyl-2-butenal | 0.023 |
| 19.61 | heptanoic acid | 0.331 |
| 19.70 | Iso butyl laurate | 0.054 |
| 19.73 | p-Methylguaiacol | 0.022 |
| 19.87 | Maltol | 0.037 |
| 19.89 | 2-Acetylpyrrole | 0.015 |
| 19.93 | Delta-octalactone | 0.185 |
| 20.08 | Isoquinoline | 0.055 |
| 20.22 | Tetradecanoic acid, methyl ester | 0.192 |
| 20.32 | 4- tert-butylaniline | 0.072 |
| 20.45 | 1H-Pyrrole-2-carboxaldehyde | 0.069 |
| 20.52 | Gamma-nonalactone | 0.155 |
| 20.62 | Tetradecanoic acid, ethyl ester | 0.737 |
| 20.69 | Octanoic acid | 3.83 |
| 21.45 | Propyl tetradecanoate | 0.073 |
| 21.69 | gamma-Decalactone | 0.016 |
| 21.76 | Nonanoic acid | 0.492 |
| 22.18 | 4-Vinyl-2-methoxy-phenol | 0.101 |
| 22.37 | Hexadecanoic acid, methyl ester | 0.259 |
| 22.84 | Hexadecanoic acid, ethyl ester | 0.711 |
| 23.05 | Decanoic acid | 1.119 |
| 23.11 | Ethyl 9-hexadecanoate | 0.067 |
| 23.22 | 1-Acetoxy-2,3-dihydroxypropane | 0.100 |
| 23.56 | Methyl phthalate | 0.040 |
| 23.82 | Glycerol | 4.96 |
| 23.99 | 2,5-Dihydrothiophene | 0.714 |
| 24.87 | 2,3-Dihydro-thiophene | 0.327 |
| 25.67 | Benzoic acid | 0.047 |
| 26.02 | 9-Octadecenoic acid, methyl ester | 0.248 |
| 26.27 | Octadecanoic acid, ethyl ester | 0.029 |
| 26.62 | (Z)-9-Octadecenoic acid , ethyl ester | 0.938 |
| 26.81 | (Z,Z)-9,12-Octadecadienoic acid, methyl ester | 0.026 |
| 26.89 | 5-Hydroxymethylfurfural | 0.031 |
| 27.23 | (Z,Z)-9,12-Octadecadienoic acid , ethyl ester | 0.050 |
| 27.37 | Isobutyl phthalate | 0.087 |
| 28.91 | Tetradecanoic acid | 0.179 |
| 29.17 | Oleic acid | 0.556 |
| 31.65 | Hexadecanoic acid | 1.10 |
